# A new detection algorithm for alien intrusion on highway

**DOI:** 10.1038/s41598-023-37686-w

**Published:** 2023-07-01

**Authors:** Junmei Guo, Haitong Lou, Haonan Chen, Haiying Liu, Jason Gu, Lingyun Bi, Xuehu Duan

**Affiliations:** 1 The School of Information and Automation Engineering, Qilu University of Technology (Shandong Academy of Sciences), Shandong, China; 2 The School of Electrical and Computer Engineering, Dalhousie University, Halifax, Canada

**Keywords:** Engineering, Electrical and electronic engineering

## Abstract

In recent years, highway accidents occur frequently, the main reason is that there is always foreign body invasion on the highway, which makes people unable to respond to emergencies in time. In order to reduce the occurrence of highway incidents, an object detection algorithm for highway intrusion was proposed in this paper. Firstly, a new feature extraction module was proposed to better preserve the main information. Secondly, a new feature fusion method was proposed to improve the accuracy of object detection. Finally, a lightweight method was proposed to reduce the computational complexity. We compare the algorithm in this paper with existing algorithms, the experimental results showed that: On the Visdrone dataset (small size targets), (a) the CS-YOLO was 3.6% more accurate than the YOLO v8. (b) The CS-YOLO was 1.2% more accurate than the YOLO v8 on the Tinypersons dataset (minimal size targets). (c) CS-YOLO was 1.4% more accurate than YOLO v8 on VOC2007 data set (normal size).

## Introduction

**Figure 1:**
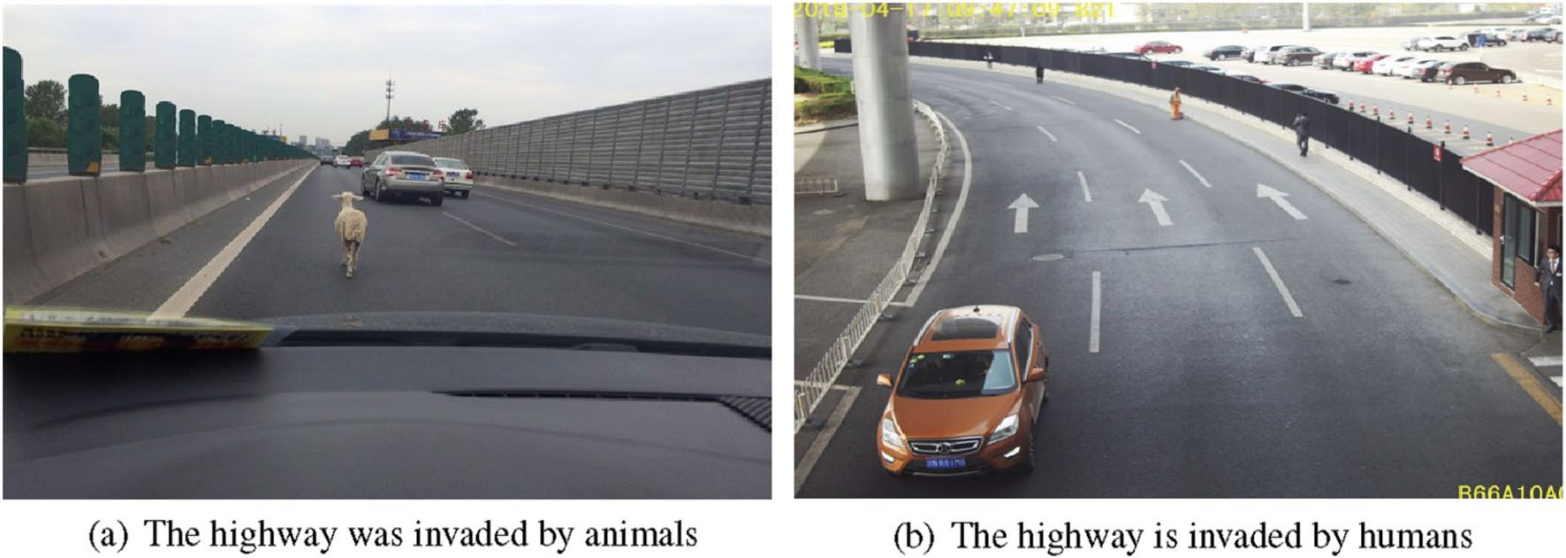
Foreign body invasion of highway.

Highway is the main component of transportation facilities, and plays a decisive role in economic development. Due to the long section of the highway and the complex terrain, the dangerous incidents on the highway occur frequently. The foreign matter invading the highway is the main threat to people’s life safety on the highway, and pedestrians and animals are the main foreign matter invading the highway. As shown in Fig. 1. Frequent foreign matter intrusion on highways seriously affects the safety of people’s lives and property, so it is very important to detect the intrusion foreign matter timely and accurately and send out alarm information for the safety of railway operation. The traditional highway monitoring system is mainly based on video surveillance^1^. Restricted by the long section of the highway, the complex environment and the limited manpower, there are some problems such as false detection and missed detection during the process of daily use.

In the past decade, with the rapid development of deep learning, object detection algorithms based on deep learning have been widely recognized in various fields^2^. The complex environment on the highway is easily affected by light and shadow changes, as well as the large number of vehicles and the subjective influence of human eyes, which makes it difficult for traditional cameras to directly capture foreign objects. Therefore, it has become a major trend to integrate the object detection algorithm based on deep learning into the highway foreign object intrusion detection technology^3–8^. In the application of object detection to the camera technology, the YOLO series algorithm is crucial. Different from the two-stage algorithm, it pays attention to the detection accuracy while ensuring real-time performance. In particular, the newly released YOLOv8 detection algorithm has high scalability, can switch between different versions of YOLO projects at will, and has strong deployment ability.

While YOLOv8 performs better, it focuses on the full range of dimensions and does not focus on size-specific target detection. So an efficient improved strategy (CS-YOLO) was proposed to solve the problem of foreign object invasion on the highway. CS-YOLO is an improved version of YOLOv8, which can more accurately identify highway foreign body invasion cases with complex scenes. The complexity of the scene mainly refers to the influence of lighting, shielding and weather. The main innovations and contributions of this paper are as follows:

(a) Based on the original network structure of YOLOv8, a novel network structure module was designed(C3D). The C2f module in front of the detector was replaced by C3D, which makes the retained information more comprehensive and steadily improves the detection accuracy.

(b) A new feature fusion method (SFPN) was proposed in this paper, which perfectly combined the shallow information and deep information, retained more original information, and effectively increased the detection accuracy of the model.

(c) A lightweight method was designed to effectively reduced the computational complexity.

This paper is mainly divided into the following sections: The second part introduces the current mainstream detectors and the main idea of YOLOv8. Section "Measures for improvement" mainly introduces the innovations of this paper. The third part focuses on the experimental results and comparative experiments. The fifth part is the summary and the future plan.

## Related works

With the gradual development and improvement of object detection and tracking algorithms based on deep learning in the past two decades, a variety of detection algorithms suitable for different scenes have emerged and gradually improved. There are two kinds of object detection methods based on deep learning, namely one-stage and two-stage. The main idea of Two-stage is to generate candidate regions first, and then perform feature extraction at last. Although its accuracy is high, its detection speed is slow. The main representative works are: Fast R-CNN, Faster R-CNN, Faster R-CNN and so on^9–12^. The main idea of one-stage is to directly extract features from images and then classify them. Its main representative works are YOLO series algorithm^3–8^, SSD series algorithm^13–15^. From their main ideas, it can be seen that one-stage algorithm is faster and more real-time, so it is more suitable for highway foreign object intrusion detection. Among the one-stage algorithms, the YOLO series of algorithms is the most prominent at present. Especially the newly released YOLOv8 in 2023, which paied attention to practice and had strong deployment ability.

After the continuous research of predecessors, there have been many foreign object intrusion detection technologies. But they all have some problems, He et al^16^proposed a two-stage algorithm for rail transit obstacle detection, which could only guarantee accuracy while giving up real-time performance, which was very undesirable for foreign object intrusion detection on highways. The high-speed rail intrusion based on YOLOv3 network proposed by Wang et al^17^ ensured the real-time performance while abandoning part of the accuracy. YOLOv8 has better performance. It combined the advantages of many YOLO series algorithms, paied attention to practice, and had high accuracy while ensuring real-time performance. Therefore, this paper took YOLOv8 as the research object to develop a foreign object intrusion detection algorithm more suitable for highway^18,19^.

Wu et al. decouples the shared features into detection-specific and ReID-specific representations. Their lightweight version runs at 26.6 FPS. The YOLO series algorithm is generally around 50 FPS^20^. Wu et al. took the approach of transforming shared features into detection-specific and ReID-specific representations. Looking for features and commonalities between the two missions, the FDN will detect and ReID collaborate in ways that make their Jupiter a state-of-the-art tracking model^21^. In order to completely meet the real-time problem of tracking, we choose the YOLO series model. Wu et al. proposed a key feature capture network (CFCN) to extract the adaptive discriminant features of each frame’s receiving field. Feature extraction network also plays a very important role in target detection^23^. At present, YOLO series model is also a relatively advanced feature extraction network model in target detection. Wu et al. propose a new pyramid fusion network (PFN) to capture pixel-by-pixel relationships of multi-level features and aggregate them into features with richer semantic information. A channel transformation enhancer (CTE) is proposed to model the dependencies between feature mapping channels and predict fine-grained identity embedding. In the task of tracking, it is divided into two parts, namely detection and re-identification?. Detection is the pre-task of tracing. So the real-time detection is very important in the tracking task. So we chose the YOLO series model which is fast.

### The network structure of YOLOv8

YOLOv8’s backbone still used the CSPDarknet53 structure, which contained multiple CSP-inspired C2f modules^17^. The convolution kernel size in front of each C2f module is 3$\times$3 with stride=2, which plays the role of downsampling. The authors of CSPNet believe that the inference computation is too high due to the repetition of gradient information in the network optimization, so the CSP module first splits the feature maps of the base layer into two parts and then merges them through the cross-stage hierarchy. This method can reduce the amount of calculation while ensuring the accuracy. At the end of backbone, the SPPF module is still used to extract features through three pooling operations to improve the receptive field of the network.

The feature fusion method used in the Neck part is still FPN+PAN. The FPN is designed to be able to solve the simultaneous detection of objects of different sizes, based on which a bottom-up PAN is added. FPN conveyed strong semantic features from the top down, while PAN conveyed strong positioning features from the bottom up. YOLOv8 combined the two to strengthen the feature fusion ability of the network and further solved the problem of multi-scale targets.

The Head part used YOLOX’s decoupling head operation to separate classification and localization, and also changed from Anchor-Based to Anchor-Free. Classification focuses on the texture content of the target, while localization focuses on the edge information of the target^24,25^. In order to avoid a large increase in calculation, YOLOv8 first performs a 1$\times$1 dimension reduction operation, and then connects two branches of classification and positioning to form a double harvest effect of detection and speed.

**Figure 2:**
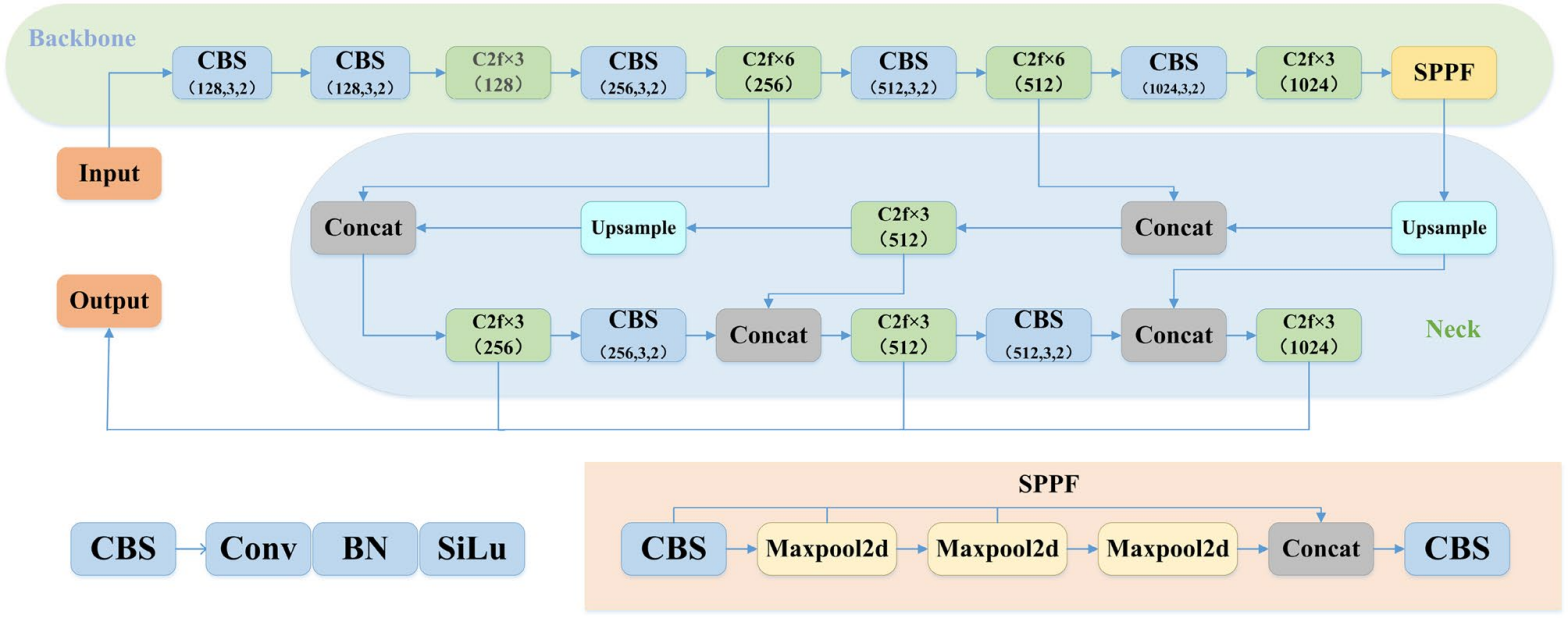
YOLOv8 network structure diagram. CBS is composed of Convolution, Batch Normalization, and SiLu activation functions. The SPPF is composed of three Maxpool tiers and two CBS.

In order to satisfy the requirements of real-time monitoring of foreign body intrusion on highways, a detection method of foreign body intrusion on highways based on YOLOv8 (CS-YOLO) was proposed in this paper. As a newly issued single-stage object detection algorithm, both the detection accuracy and real-time performance meet the needs of the project. In YOLOv8, the Backbone and Head parts are composed of multiple CBS and C2f modules. It can be seen that the strength of the feature extraction ability of YOLOv8’s network module is mainly determined by C2f. After continuous testing, it was found that there are still some problems in C2f, so this paper improved C2f under the premise of the basic framework of YOLOv8 remains unchanged. This improvement greatly reduces the false detection rate and missed detection rate of foreign body invasion on highways. Although YOLOv8 uses the feature fusion method of FPN+PAN to enrich the semantic features and positioning information, most of the original information is lost through laye-by-layer feature extraction. Therefore, the feature fusion network (SFPN) was improved by us to make the shallow information and deep information more perfect combination. Figure 2 shows the network structure of YOLOv8.

## Measures for improvement

Although YOLOv8 collects the advantages of each YOLO version, there are still many problems in the recognition of special scenes and special circumstances, especially the detection effect of small objects is not satisfactory. In view of these problems, we analyze the following points: (a) Problems such as target interference, poor viewing Angle, and environmental changes widely exist in highways, which lead to poor detection effect; (b) In complex environments such as lighting, shading, weather, and so on, objects are prone to overlap, and after multiple feature extraction, it is easy to lose a lot of edge information, resulting in a decrease in detection accuracy.

In order to solve the above problems, we proposed a new highway alien intrusion detection algorithm (C3S-YOLO). The C2f module was improved to capture more context information and retained the feature information of the object more comprehensively. An efficient feature fusion method was proposed to better fuse shallow information and deep information, and improved the accuracy of target detection in complex scenes.

### C3D module

**Figure 3:**
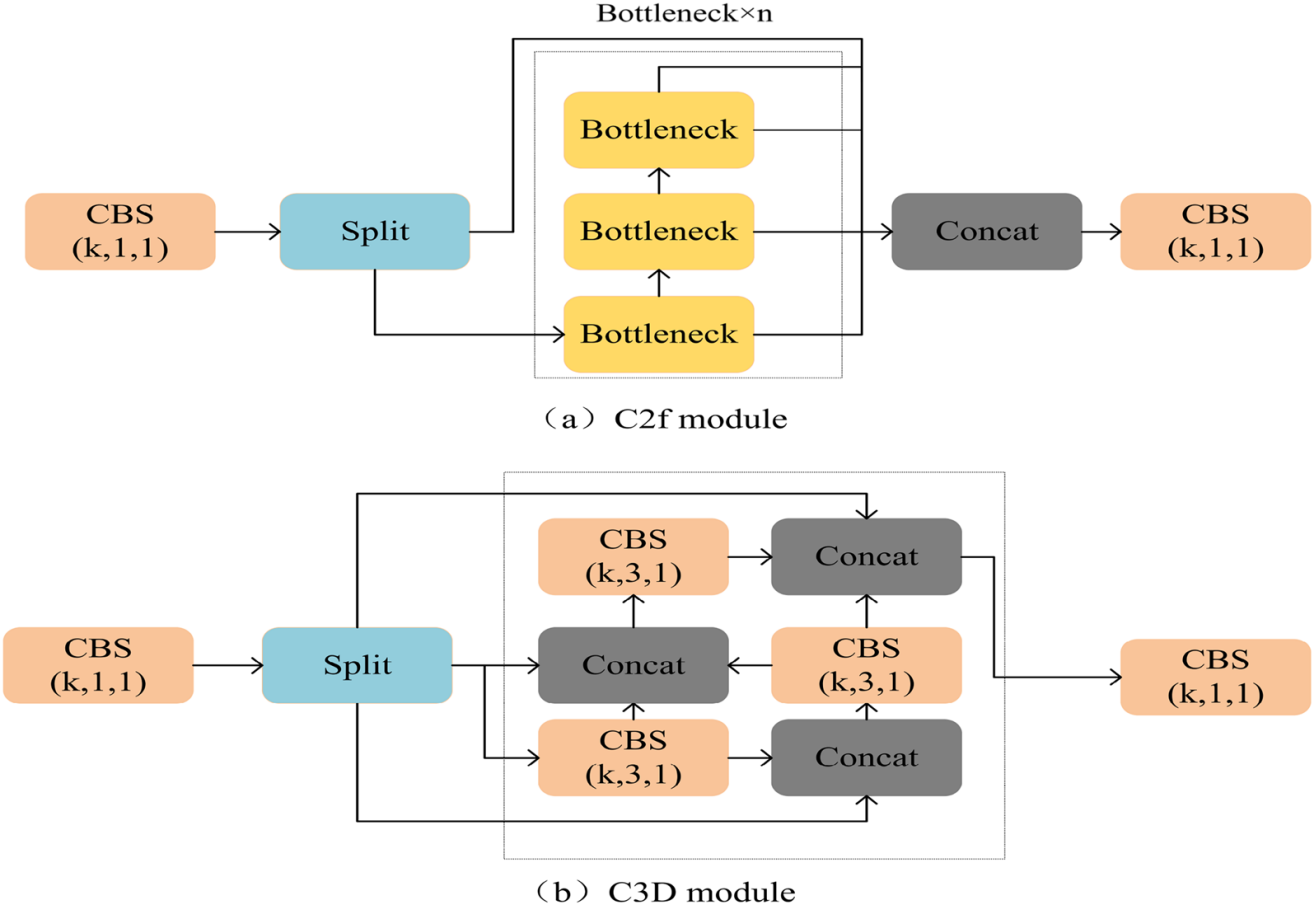
Diagram of C2f and C3D network structures.

Compared with C2f, a more efficient densely connected mechanism was proposed, which connected all layers and performs a concatenation after feature extraction of each layer. There are many module layers in C3D, and the feature maps formed by each module layer after feature extraction are of the same size and densely connected between layers^26^. The shallow network mainly focuses on texture features, while the deep network focuses on ontology features, and the information in each layer is crucial. Therefore, the method of splicing the information of each layer by this dense connection mechanism was used by us, so that the network structure could learn more complete feature information and improved the accuracy of detection. The specific structure is shown in Fig. 3.

The specific implementation details of the network are described in the following. After the convolution of each layer, K feature maps are generated, which means that the number of channels in each feature map is K. In general, a splicing is performed after each layer of convolution, however, the number of channels of the feature map formed after splicing will be larger than K, resulting in a large input of the next layer of convolution and a large amount of calculation. Therefore, before convolution, we used the convolution alignment with the size of 1$\times$1 to reduce the number of channels to K, and then performed convolution operation. Due to this connection method, the gradient backpropagation of the whole network is improved, which makes the network easier to train. The connection of each layer is fused by Concat, so the feature reuse is better realized.

The specific implementation is as follows: first, a convolution kernel of size 1$\times$1 is used for dimensionality reduction, then a convolution kernel of size 3$\times$3 is used for convolution, and finally the output and the original input information are used for Concat operation, so that the C3D module is formed by repeating three times. The specific structure is shown in Fig. 3.

### SFPN

**Figure 4:**
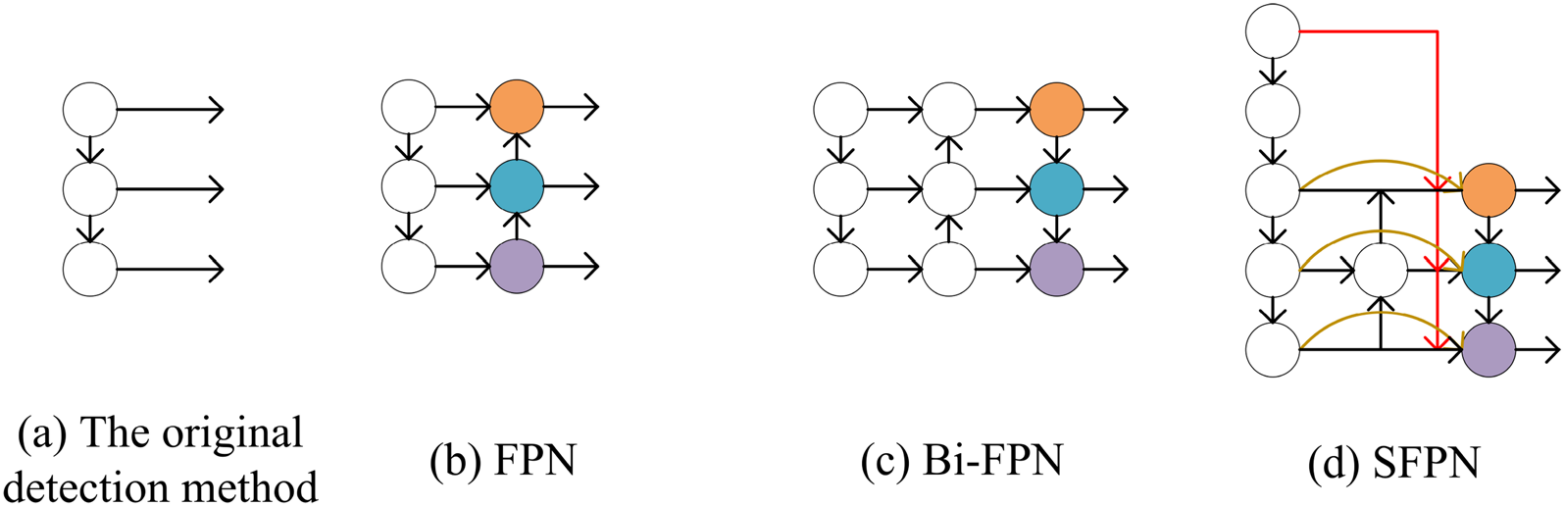
(**a**) Shows the original detection method. (**b**) Is the main idea of FPN feature fusion method. (**c**) is the main idea of BiFPN feature fusion, which is also adopted by YOLOv8. (**d**) is the SFPN proposed in this paper.

As shown in Fig. 4a, the earliest feature fusion is directly predicted after backbone feature extraction, but this prediction does not carry out feature fusion, resulting in low final detection accuracy. After continuous attempts, Lin, et al.^18^. proposed the idea of FPN as shown in Fig. 4b, which uses the fused feature layer with more semantic information to predict, and finally finds that it can improve the accuracy to a certain extent. Finally, a new feature fusion network BiFPN was proposed^27^. This is shown in Fig. 4c. It fused the shallow semantic information and the deep location information at the same time, so that the final detection accuracy was greatly improved. YOLOv8 also uses this method. However, we found in our experiments that the information retained by BiFPN feature fusion method is not complete, and the shallower position information is missing. Therefore, this SFPN feature fusion method was proposed in this paper, which perfectly fused the shallowest information and the deep information. The specific structure is shown in Fig. 4d.

Through the comparison in Fig. 4, we can see that the network has carried out multiple down-sampling operations during the feature extraction, which also leads to serious information loss in the final part of the feature fusion, and such shortcomings will be more and more obvious. Most researchers have realized this problem, however, their way to deal with it is to add a fourth detection layer, but this method has a big drawback, that is, it increases a lot of calculation. Finally, the purpose of real-time performance cannot be achieved. Therefore, in this paper, the first layer was directly downsampled and fused into BiFPN, which not only improved the detection accuracy, but also did not increase the amount of calculation. The specific operation is as follows: Maxpool2d is used to perform three consecutive down-sampling operations on the first layer, and then each layer is fused with its own feature layer of corresponding size for final detection.

### Lightweight implementation

In order to further achieve lightweight, this paper uses the GoogLeNet method^28^. Convolutions of size 1$\times$1 stacking more convolutions in the receptive field of the same size can extract richer features. When we do a normal convolution (3$\times$3) and then another convolution (1$\times$1), it is equivalent to doing a fully connected computation on all the features for that pixel. So the concatenation of two convolutions can combine more nonlinear features.

If we use a convolution of size 1$\times$1 to reduce the dimensionality before the above operation, we can reduce the computational complexity. When a convolutional layer has a large number of input features, the convolution operation on this input will produce a huge amount of calculation. However, if the dimension of the input is reduced first and the number of features is reduced before the convolution, the calculation will be significantly reduced. Through many experiments, it was proved that the dimension reduction with the size of 1$\times$1 convolution did not affect the final training results, and its effect was equivalent to a compression of the original feature map, and did not affect the final training results.

**Figure 5:**
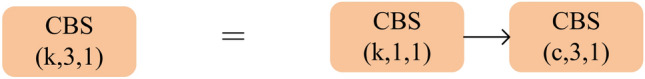
Lightweight conversion method.

Therefore, we will use the convolution (3$\times$3) of the whole paper to reduce the dimension first by using the convolution of size 1$\times$1, then use the convolution of size 3 difference 3 for convolution operation, and finally use the convolution of size 1$\times$1 for dimension increase. The specific structure is shown in the Fig. 5.

At the same time, we removed the large size target detector of the detector, which made our model more lightweight and did not affect the detection effect of small size targets.

Using the lightweight approach resulted in a 26% reduction in GFLOPs.

**Figure 6:**
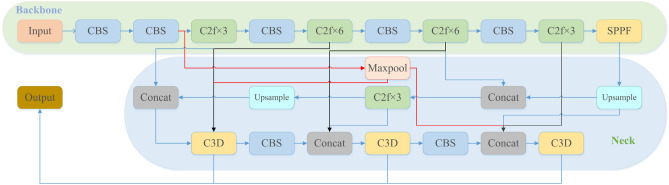
CS-YOLO network structure diagram.

Figure 6 shows the overall network structure diagram of CS-YOLO.

## Experiment

In order to verify that each stage of improvement in this paper could achieve better results in foreign body intrusion detection on highways, the ablation experiments was tested on the Visdrone data set and compared it with YOLOv8. In order to verify that this algorithm could achieve better results in different environments or different goals, this paper conducted experiments on PASCAL VOC2007 dataset and Tinyperson dataset respectively. These two datasets are used for comparison experiments because the targets represented by these two datasets are normal-size targets and small-size targets, respectively. Finally, we selected several pictures of foreign body invasion on highways for actual effect detection.

According to the experience and conclusions of YOLO series algorithms and the memory usage of GPU, we set the batch size to 4 and epochs to 200 (it is known from many experiments that the experiment converges at 120 rounds). We set it to 0.01.

### Experimental platform

The system used for the experiment in this paper is Windows11, and the system hardware facilities: 16G RAM, NVIDIA GTX3070 GPU, Intel i512400f CPU. Software platform: torch 1.12.1+cu113, Anaconda.

### Valuation index

Evaluation metrics: Mean average precision (mAP), average precision (AP), precision (P), recall (R) and F1 score. The formulas for P and R are as follows:

1 $$\begin{aligned} P= & {} \frac{{TP}}{{(TP + FP)}} \end{aligned}$$

2 $$\begin{aligned} R= & {} \frac{{TP}}{{\left( {TP + FN} \right) }} \end{aligned}$$

TP is the number of correctly predicted bounding boxes, FP is the number of incorrectly judged positive samples, and FN is the number of undetected targets.

Average Precision (AP) is the average accuracy of the model. mean Average Precision (mAP) is the average value of the AP. K is the number of categories. F1 Score, also known as the balanced Score, is defined as the harmonic average of accuracy and recall. The formulas for AP, mAP and F1 Score are as follows:

3 $$\begin{aligned} AP= & {} \int _0^1 {p(r)dr} \end{aligned}$$

4 $$\begin{aligned} mAP= & {} \frac{1}{k}\sum \limits _{i = 1}^k {A{P_i}} \end{aligned}$$

5 $$\begin{aligned} F1= & {} \frac{{2\mathrm{{PR}}}}{{P + R}} \end{aligned}$$

### Datasets

In order to verify the universality of our model, the data sets we selected were all publicly available general data sets.

The data set for ablation experiments in this paper is Visdrone data set, which is the main representative of human or animal foreign body invasion on highways. Long-distance small-size target detection is often required for real-time detection and avoidance on highways. So we chose Visdrone data set as the main test data set in this paper.

The VisDrone dataset was collected by the AISKYEYE team of the Machine Learning and Data Mining Laboratory of Tianjin University. All benchmark datasets were captured by drones, including 288 video clips, including a total of 261908 frames and 10209 still images. These frames consist of more than 2.6 million manually annotated boxes of commonly used targets.

PASCAL VOC2007 dataset and Tinyperson dataset were used for comparison experiments. The PASCAL VOC2007 data set is one of the necessary data sets to verify the effectiveness of this method in highway foreign body intrusion. There are 20 classes in this dataset, which contains 11530 images for training and validation, and 27450 calibrations for the region of interest. PASCAL VOC2007 provides an excellent set of standardized datasets for image recognition and classification. It has annotations in each image, and the labeled objects include 20 categories including people, animals (such as cats, dogs, birds, etc.), transportation vehicles (such as cars, boats, airplanes, etc.), furniture (such as chairs, tables, sofas, etc.). Each image has an average of 2.4 objects. There are two categories in TinyPerson data set, sea person and earth person. This data set is mainly for verification of small targets, and the category is only human, which can perfectly verify that the algorithm also has good experimental effect in small size targets.

### Ablation experiment

In order to verify that these improvements in each stage of this paper were effective, we conducted ablation experiments on the Visdrone dataset and compared it with YOLOv8 at each stage. The Visdrone data set also includes different scenes, weather and light. There are many targets in complex environments, and a large number of images have common visibility and occlusion conditions. It meets all the requirements of foreign body invasion on highways, so this paper chooses to use the Visdrone data set as the evaluation basis for ablation experiments.

In order to show the experimental data clearly, map0.5, MAP0.5:0.9, P and R are used as evaluation indexes in this experiment, and the experimental results are shown in Table 1.

**Table 1:** Ablation experiment.

Detection algorithm	Module			Result				
	C3D	Feature fusion	lightweight	mAP0.5	mAP0.5:0.95	P	R	F1
YOLOv8				39	23.2	50.8	38	43.5
CS-YOLO	$\checkmark$			41.3	24.9	52	39.7	45
CS-YOLO	$\checkmark$	$\checkmark$		42.6	25.7	53.4	40.7	46.2
CS-YOLO	$\checkmark$	$\checkmark$	$\checkmark$	42.5	25.7	53.6	40.7	46.3

It can be seen from the table that for the detection of small-size targets in complex scenes, the improved algorithm has a certain improvement in each stage. And the recall rate is improved by 2.7%, which determines that there is a lot of room for improvement. It can be proved that the three directions improved in this experiment are obviously effective: (a) C3D module effectively solves the problem of losing a lot of important information due to being misled by large-size objects in the feature extraction process. (b) The improved feature fusion method effectively improves the target detection accuracy. (c) The proposal of lightweight method effectively realizes the purpose of reducing the number of parameters. The experimental results show that the improvement of the algorithm in each stage can improve the learning ability of the model.

**Figure 7:**
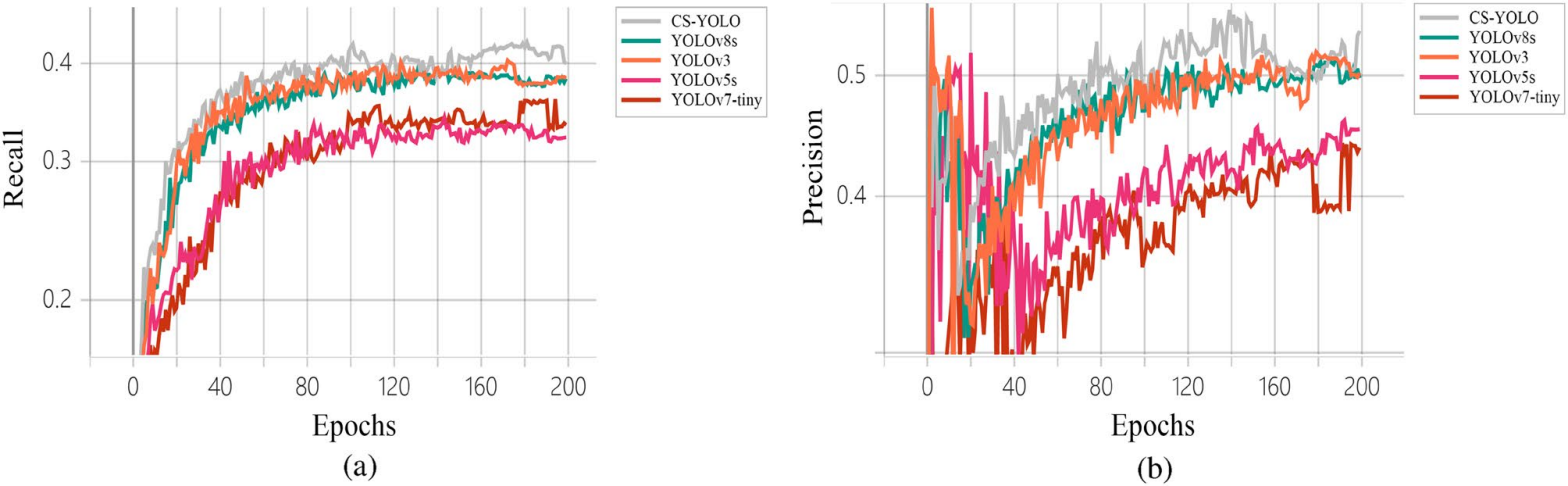
Results comparison chart. (**a**) Precision, (**b**) Recall.

In order to see the changes of P and R between different algorithms more intuitively, we draw P and R curves (Fig. 7).

We analyzed the main foreign bodies (human and animal) on the highway. In Fig. 7a, we can see that the detection accuracy of both humans and animals reaches about 80. Such detection accuracy is very high. Therefore, it can be proved that our detection effect is effective.

### Controlled experiments with different data sets

PASCAL VOC2012 dataset and Tingperson dataset were used by us to do control experiments. The reason for choosing PASCAL VOC2012 data set is that there are a wide range of categories in this data set, including six types of animal. Foreign body invasion on highways mainly refers to humans and animals, so we choose this data set as the first set of comparison experiments. The reason for choosing the Tinyperson dataset is that people need to detect foreign objects in advance on the highway, which means that we need to make accurate judgments from a distance, so we need to make more accurate detection of small size targets. Tinyperson only contains two categories, and both are very small size targets, which meets the needs of this project. We select YOLOv8 and CS-YOLO with the highest weights for verification as shown in the Table 2.

**Table 2:** Comparison of CS-YOLOH and various algorithms on different data sets.

Detection algorithm	Dataset			Result			
	Visdrone	VOC	Tinyperson	mAP0.5	mAP0.5:0.95	FLOPs(G)	FPS
YOLOv3	$\checkmark$			38.8	21.6	283.8	36.36
YOLOv5s	$\checkmark$			38.1	21.7	15.8	57.8
YOLOv7-tiny	$\checkmark$			30.7	20.4	13.1	125
YOLOv8	$\checkmark$			39	23.2	28.6	357
CS-YOLOv8	$\checkmark$			42.6	25.7	42.3	192.3
YOLOv3		$\checkmark$		84.1	53.1	283.8	64.5
YOLOv5s		$\checkmark$		78	51.6	15.8	322.6
YOLOv7-tiny		$\checkmark$		69.1	42.4	13.1	384.6
YOLOv8		$\checkmark$		83.1	63	28.6	370.4
CS-YOLOv8		$\checkmark$		84.5	64.4	42.3	256.4
YOLOv3			$\checkmark$	18.5	5.79	283.8	52.36
YOLOv5			$\checkmark$	18.3	5.81	15.8	147.1
YOLOv7-tiny			$\checkmark$	16.9	5.00	13.1	192.3
YOLOv8			$\checkmark$	18.1	6.59	28.6	222.2
CS-YOLOv8			$\checkmark$	19.3	7.32	42.3	224.7

In Table 2, we set a total of four metrics. mAP0.5, mAP0.95, GFLOPs, FPS. FPS represents the detection speed. The higher the FPS, the faster the detection speed. GFLOPs represents the model complexity. As can be seen from the Table 2, other detection algorithms only have advantages in a single index. For example, YOLOv8’s FPS is very high, but its mAP is 3.6% lower than CS-YOLO, which is a non-negligible data. Therefore, in terms of overall evaluation metrics, our model achieves a better balance between mAP and FPS.

**Figure 8:**
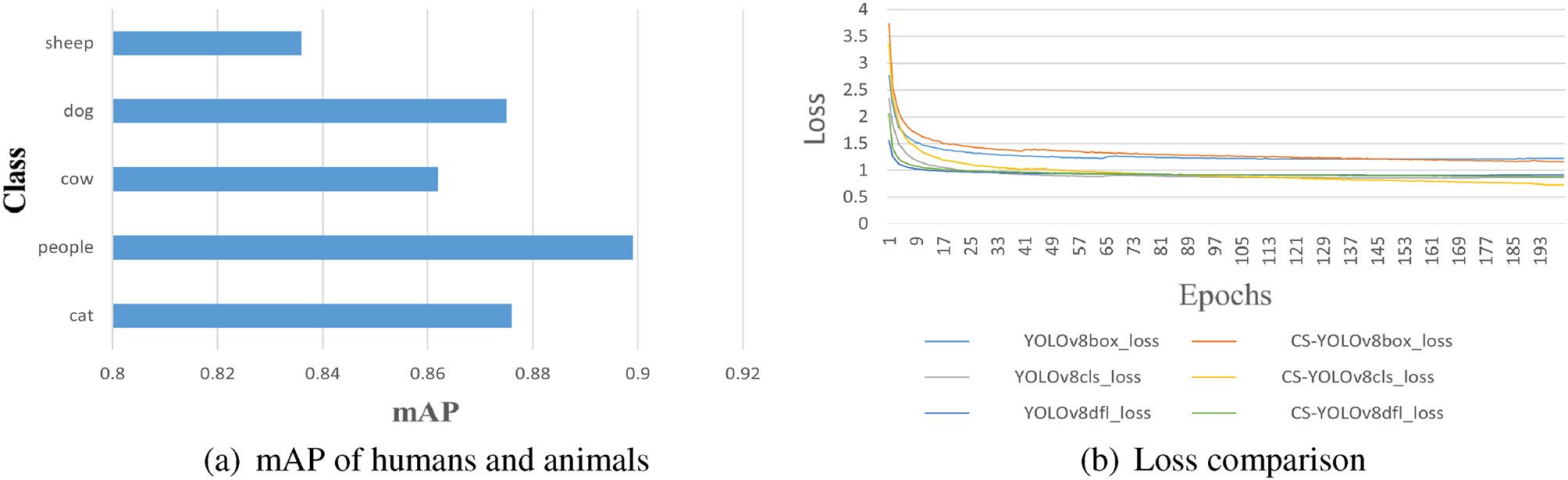
Class contrast and loss contrast.

As we can see from Table 2, CS-YOLO has the highest detection accuracy under each data set, especially in the Visdrone data set, the improvement is obvious. The target size of Visdrone dataset is consistent with the size of foreign body intrusion on highways, so CS-YOLO is more suitable for foreign body intrusion detection on highways. From Fig. 8b, we can see the main information of loss in CS-YOLO.

**Figure 9:**
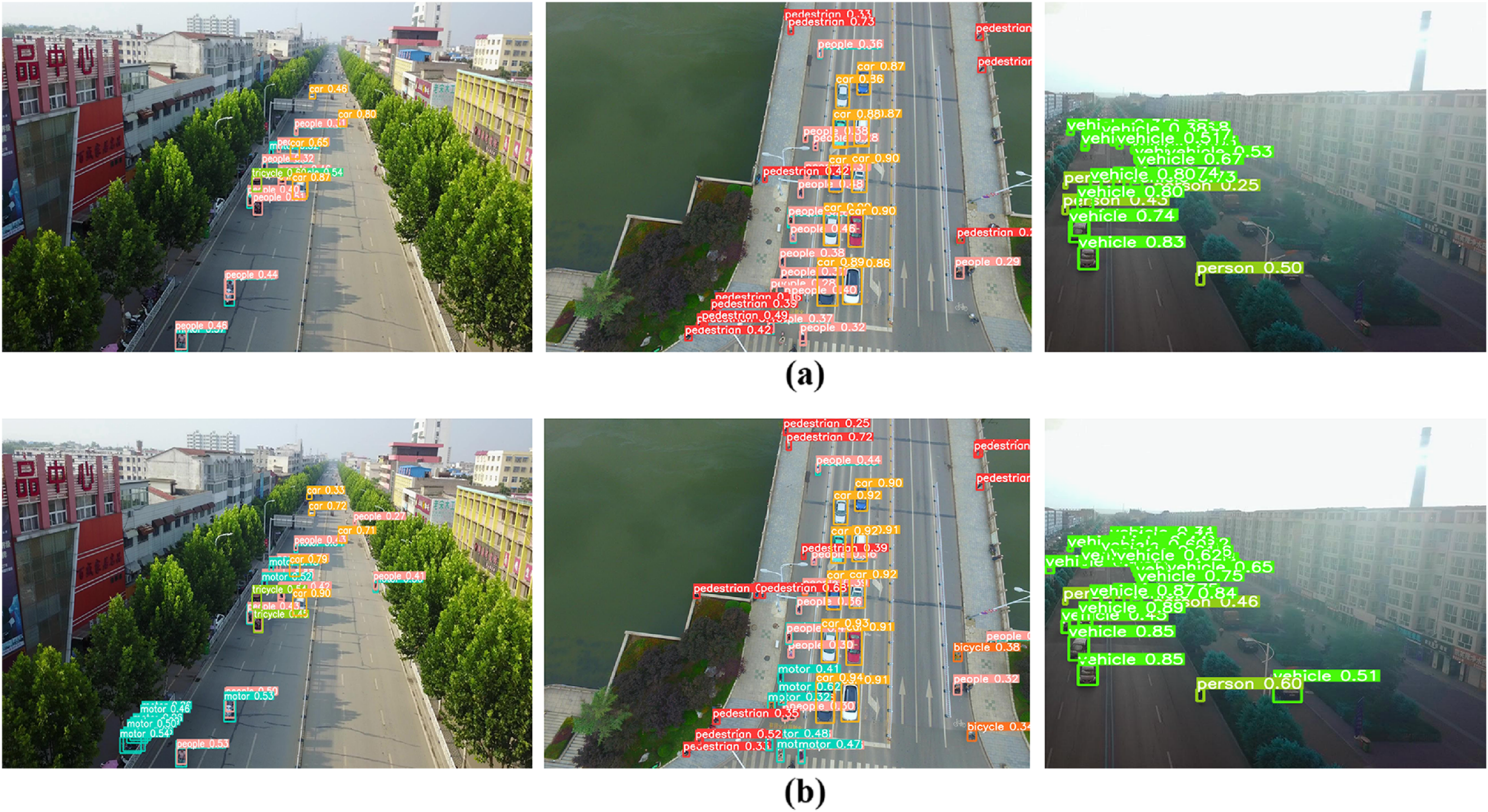
Comparison of YOLOv8 and CS-YOLO experiment, (**a**) is the detection result of YOLOv8. (**b**) is the detection result of CS-YOLO.

From the results, it can be seen that the algorithm proposed in this paper has a strong feature fusion ability, which can better use the shallow position information and the semantic information in the deep feature map. Through the dense link mechanism, the detection information of the model for each object is improved.

In order to see more directly that our algorithm is better for small size targets, we choose a group of pictures taken by the UAV. From Fig. 9, we can clearly see that YOLOv8 has a lot of missed detection cases for smaller targets, while CS-YOLO has far fewer missed cases than YOLOv8. As can be seen from this group of comparison figures, CS-YOLO is a foreign body intrusion detection technology that can be applied to highways.

## Conclusions

In order to reduce accidents caused by foreign body invasion on the highway. In this paper, the problem of highway intrusion detection was deeply explored, and the object detection algorithm based on deep learning (YOLOv8) was applied to the highway camera. However, it was found that YOLOv8 is not ideal in the experiment, so this paper proposed a new algorithm CS-YOLO and applies it to the camera. A dense connection mechanism was proposed to achieve better feature reuse, a new feature fusion method was proposed to improve the detection accuracy, and a lightweight method was proposed to reduce the computational complexity. This paper mainly focuses on the innovation carried out on the network structure, and we will continue our in-depth research in this direction next.

## Data Availability

All the images and experimental test images in this paper were from the open source VisDrone dataset,PASCAL VOC dataset and Tinyperson dataset. These datasets analyzed during the current research period can be found at the following website. Visdrone: https://github.com/VisDrone/VisDrone-Dataset. PASCAL VOC2007: http://host.robots.ox.ac.uk/pascal/VOC/voc2007/. Tinyperson: m6z.cn/6vqF3T.
